# Authors Response: Preeclampsia prevention: a case-control study nested in a cohort

**Published:** 2016-03-30

**Authors:** Alberto Alzate, Rodolfo Herrera, Lucia Maracelly Pineda

**Affiliations:** 1 Grupo de Investigación en Epidemiología y Servicios (GRIEPI). Universidad Libre-seccional Cali, Cali, Colombia; 2 Coomeva EPS. Cali, Colombia

## To the Editor:

When Bautista 1 affirm that "none of the cases in 13-18 year old women was treated with CC+CLA" there is a misinterpretation of the fourfold table in case control studies. What you can read in the four fold table for age 13 to 18 Table A

**Table A.** Frequency of exposed and unexposed women

Exposition	Preeclampsia	Controls
Exposed (CC+CLA)	0	29
Unexposed (CC)	49	150

Meaning that only 29 primigravidae received CC+CLA and that among them there was not any case of preeclampsia, becoming all the exposed controls. Remember that we are working with incident cases in a nested case control design. Their odds of becoming a "case" are 0/49 compared to the odds of 29/150 of becoming a "control". And the sample size is bigger enough to assess an OR with a 95% confidence interval between 0.00 and 0.44. The purpose of the authors in publishing the paper was to ask to the scientific community what is happening in this age group that we don't know, and why is Calcium still recommended despite of the alarming perspective of no effect and increasing incidence rates of preeclampsia.

With respect to the apparent discrepancy between Tables 2 and 3 (original article) 2, it is easy to see that the sum of cases and controls before 2013, during the only calcium period (Table B):

**Table B.** First period before 2013.

Treatment CC	Exposition	Preeclampsia	Controls	Total
13-18	Exposed (CC)	13	40	
Unexposed (CC)	28	115	196
19-34	Exposed (CC)	82	244	
Unexposed (CC)	174	489	989
35-45	Exposed (CC)	11	26	
Unexposed (CC)	20	20	77
		328	934	1,262

Whereas the table when all cases and controls were evaluated after the introduction of the administration of calcium citrate plus conjugated linoleic acid during the second period (2013-2014) in Table 3 (original article) (Table C). 

**Table C.** Second period (2013-2014)

Treatment CC+CLA	Exposition	Preeclampsia	Controls	Total
13-18	Exposed (CC+CLA)	0	29	
Unexposed (CC+CLA)	49	150	228
19-34	Exposed (CC+CLA)	57	131	
Unexposed (CC+CLA)	244	696	1,128
35-45	Exposed (CC+CLA)	4	3	
Unexposed (CC+CLA)	33	45	85
		387	1,054	1,441

This is because during 2013, 59 new cases and 110 controls were recruited into the study. There is no way to sum the 1,441 + 1,262, and nowhere it is suggested in the paper.

With respect to Monteverde, Coronel-Acosta and Segura letter 3, the 4.5% prevalence mentioned by them is the prevalence in Villavicencio, Colombia in 2004. The proportion of new cases among primigravidae of medium and high class income, privately insured, in Cali, Colombia between 2010 and 2014 was 10%, and the risk in primigravidae is always higher than in other pregnant women. The nested case control is recommended in situation when you have 387 incident cases in the cohort and a pool of 3,866 possible controls and, instead of searching 3,866 clinical histories, which takes about half an hour for each clinical history in our electronic records, it is cost saving and equally effective to pick up randomly 1,054 controls. 

Obviously case control studies are not clinical trials, like our correspondents pointed out, and its role in the evaluation is to assess the safety and effectiveness in clinical care, using like in our case the information available in clinical histories in insurance funds. It is clear that the result is not casual ("fortuito"), when the odds ratio is cero (OR= 0.00) with 0.05% confidence intervals between 0.0 and 0.44 in adolescent primigravidae as mentioned above.
